# “The Physician in Relation to the Homeopath” in Practice

**Published:** 1903-11

**Authors:** A. N. Perkins

**Affiliations:** Sabine Pass, Texas


### "The Physician in Relation to the Homeopath" in Practice.

[The following letter from Dr. A. N. Perkins, "The Sage of Sabine," one of the fathers in Texas medicine, I publish with pleasure. The doctor takes strong issue with the editor of the "Red-Back,"—and that's something I like; confound a fellow who always agree with you,—there's never a chance for a scrap. I'm going to "let it go at that," however, this time.—DANIEL.]

SABINE PASS, TEXAS, October 17, 1903.

*F. E. Daniel, M. D., Editor Texas Medical Journal, Austin, Texas.*

DEAR DOCTOR: I have just read your editorial on the physician and the homeopath, and principles of ethics, substituted for the old code.

While I recognize you as one of our ablest medical writers, and greatly admire you as a high-toned gentleman, I hope you will pardon me when I say I think sometimes your hatred of sectarianism, especially your prejudice against the homeopath sect, causes you to take extreme positions and subjects you to the charge of being illiberal. Take, for instance, the supposed consultation: "Two homeopathic physicians are up against a breech presentation, and confess their inability to deliver. The family send for two physicians; one gives chloroform, the other delivers, and the family elects to retain the homeopath in charge."

"The physicians are dismissed," you say, on a case of this kind. "The physicians did right in delivering the woman, but they ought to have demanded, before attending the case, the dismissal of the homeos, who has confessed their inability to deliver." I do not agree with you, and I do not believe a majority of intelligent physicians will sustain you.

In the first place, the educated homeopathic physician has no exclusive system of obstetrics. He recognizes the same mechanism of labor that the regular physician does; he is taught the same principles. In the second place, when a physician asks for a consultation, he does not by so doing confess his ignorance nor does he confess his inferiority to the man with whom he wishes to consult; he only asks for assistance, oftentimes satisfied the case is progressing well. The consultation is desired to satisfy the anxiety of the family and friends.

I think in a case of this kind there should be no drawing of lines, but a recognition of each other as members of the medical profession, devoted to the alleviation of human suffering. It may,

I think, under some circumstances, be proper for a regular physician and a homeopath to meet in consultation to determine a diagnosis.

There should be peace and harmony among men devoted to the alleviation of the sufferings of humanity.

Yours truly,

A. N. PERKINS, M. D.

